# Dynamics of Foliar Responses to O_3_ Stress as a Function of Phytotoxic O_3_ Dose in Hybrid Poplar

**DOI:** 10.3389/fpls.2021.679852

**Published:** 2021-06-28

**Authors:** Benjamin Turc, Pierre Vollenweider, Didier Le Thiec, Anthony Gandin, Marcus Schaub, Mireille Cabané, Yves Jolivet

**Affiliations:** ^1^University of Lorraine, AgroParisTech, INRAE, SILVA, Nancy, France; ^2^Section Forest Dynamics, Swiss Federal Institute for Forest, Snow and Landscape Research WSL, Birmensdorf, Switzerland

**Keywords:** ozone, poplar, hypersensitive response-like, accelerated cell senescence, foliar response

## Abstract

With background concentrations having reached phytotoxic levels during the last century, tropospheric ozone (O_3_) has become a key climate change agent, counteracting carbon sequestration by forest ecosystems. One of the main knowledge gaps for implementing the recent O_3_ flux-based critical levels (CLs) concerns the assessment of effective O_3_ dose leading to adverse effects in plants. In this study, we investigate the dynamics of physiological, structural, and morphological responses induced by two levels of O_3_ exposure (80 and 100 ppb) in the foliage of hybrid poplar, as a function of phytotoxic O_3_ dose (POD_0_) and foliar developmental stage. After a latency period driven by foliar ontological development, the gas exchanges and chlorophyll content decreased with higher POD_0_ monotonically. Hypersensitive response-like lesions appeared early during exposure and showed sigmoidal-like dynamics, varying according to leaf age. At current POD_1_SPEC_ CL, notwithstanding the aforementioned reactions and initial visible injury to foliage, the treated poplars had still not shown any growth or biomass reduction. Hence, this study demonstrates the development of a complex syndrome of early reactions below the flux-based CL, with response dynamics closely determined by the foliar ontological stage and environmental conditions. General agreement with patterns observed in the field appears indicative of early O_3_ impacts on processes relevant, e.g., biodiversity ecosystem services before those of economic significance – i.e., wood production, as targeted by flux-based CL.

## Introduction

The ground-level concentrations of ozone (O_3_) have increased during the past century (Maas and Grennfelt, 2016), and are predicted to remain stable or increase during the 21st century (Revell et al., 2015; Fu and Tian, 2019). They have already reached levels negatively affecting crop plants and the natural vegetation (Wittig et al., 2009; Jolivet et al., 2016; Proietti et al., 2016; Li et al., 2017), and steady or increasing impacts are expected over the course of next decades (Karlsson et al., 2017).

Once entering the leaf through stomata, O_3_ degradation causes the formation of reactive oxygen species (ROS), the accumulation of which triggers rapid oxidative bursts (Schraudner et al., 1998; Pasqualini et al., 2003; Moura et al., 2018). ROS can also act as elicitors of programed cell death (PCD) reminiscent of plant responses during defensive plant/pathogen interactions which are subsequently designated as hypersensitive response-like (HR-like; Vollenweider et al., 2002; Bhattacharjee, 2005; Günthardt-Goerg and Vollenweider, 2007; Moura et al., 2018). In parallel, an acceleration of cell senescence (ACS), with distinct apparent mechanisms, can be observed (Pell et al., 1999; Günthardt-Goerg and Vollenweider, 2007; Vollenweider et al., 2019). The characteristic symptoms thus include marked degenerative injuries in chloroplasts, in apparent relation to an increase in the constitutive ROS load resulting from the daily photosynthetic activity. As a consequence, these latter organelles are particularly sensitive to O_3_ stress (Joo et al., 2005; Kangasjarvi et al., 2005). However, the sequence of plant reactions in response to O_3_ stress remains unclear, especially given the driving – but still partially understood – effects of interacting environmental conditions and ontological development. In field vs. climate chamber conditions, for example, the high vs. low-intensity illumination can lead to contrasted symptom expression, with clear synergies between photooxidative and O_3_ stress in the former case only (Günthardt-Goerg and Vollenweider, 2007; Paoletti et al., 2009; Moura et al., 2018; Vollenweider et al., 2019). Hence, the dynamics of responses to O_3_ stress as a function of environmental conditions needs further research.

Although the effects of O_3_ stress have been observed in both mature and developing foliage, their intensity is strongly related to the leaf ontology, the mature leaves being more sensitive than those still in expansion. However, the younger vs. older leaves can show higher rates of stomatal conductance and O_3_ uptake (Reich, 1983; Strohm et al., 1999; Bagard et al., 2008; Zhang et al., 2010; Guerrero et al., 2013), suggesting an enhanced detoxification capacity (Bellini and De Tullio, 2019). Still, the mechanisms underlying the higher O_3_ tolerance in developing foliage remain largely obscure (Strohm et al., 2002) and the differences in response dynamics as a function of leaf ontogenetic development require further investigations.

To assess and prevent O_3_ injury on vegetation and forest trees, a concentration-based index, namely, the accumulated O_3_ exposure threshold over 40 ppb (AOT40), was initially proposed (Fuhrer et al., 1997). Given the dependency of O_3_ phytotoxicity on stomatal conductance, the biologically (Karlsson et al., 2007; Mills et al., 2011) and environmentally (Musselman et al., 2006; Dizengremel et al., 2013; Büker et al., 2015) more relevant flux-based approach has been increasingly implemented. Nowadays, the O_3_ critical level (CL) is defined for given vegetation types or plant species and calculated as the Phytotoxic O_3_ Dose over a Y threshold for a specific species or group of species (POD_Y_SPEC_ (Mills et al., 2017). Based on empirical evidence from risk assessment studies – linking POD_Y_SPEC_ values to tree biomass loss or foliar injury – the current CL typically targets 4% maximum, i.e., growth reductions by oxidative stress. However, such markers represent some late O_3_ stress effects, at least partly resulting from earlier processes in foliage (i.e., reduced physiological activity/extensive cellular injury) which dynamics primarily depends on detoxification processes (Dghim et al., 2013; Dumont et al., 2014; Dusart et al., 2019a). With a view to the larger implementation and acceptance of flux-based approach, there is then an important knowledge gap regarding the dynamics and effective POD_x_ of first effective O_3_ stress effects, prior to the appearance of current risk assessment markers.

In this study, our main objective was to characterize the dynamics of early physiological and structural responses to O_3_ stress in poplar trees as a function of flux-based O_3_ dose and before, e.g., growth reduction and extensive foliar injury, the primary markers of O_3_ stress for defining O_3_ CL (Sanz and Catalayud, 2011; Mills et al., 2017). The tested hypotheses (H) included: (H1) the development of injury and growth response to O_3_ stress, as well as physiological and structural changes, proceeds in sequential order, with each response showing specific dynamics; (H2a) O_3_ elicits different injury responses within the foliage of trees (H2b) with ACS occurring before the development of HR-like lesions (Vollenweider et al., 2019); (H3a) at comparable O_3_ dose and irrespective of the applied O_3_ concentration, leaves show similar responses and (H3b) response dynamics; (H4) the dynamics of responses depends on the leaf developmental stage (Moura et al., 2018). Therefore, rooted cuttings of hybrid poplar (*Populus tremula x alba*) were exposed to three O_3_ concentrations in fully controlled conditions for a month. The leaf physiology, development of ACS and HR-like lesions, and appearance of visible injuries were monitored over the course of 29 days. The interaction between foliar response dynamics and leaf ontological development was evaluated by assessing the responses to treatments at two distinct leaf positions.

## Materials and Methods

### Plant Material and Controlled O_3_ Exposure

Young trees from a hybrid *Populus tremula x alba* clone (INRAE 717-1b4) were cultivated similarly to Cabane et al. (2004). Before the experimental exposure, micro propagated cuttings were grown for 2 weeks in 0.5 L pots containing compost (Gramoflor Universel) and perlite [1:1 (v/v)], and placed in containers covered with transparent acrylic hoods inside a growth chamber. The environmental conditions were set at 22°C/18°C day/night temperature, 350 μmol m^−2^ s^−1^ photosynthetic active radiation (PAR, 1 m below lamps) during a 14-h photoperiod (Philips Son-T Agro 400 W lamps), 75%/85% relative humidity (day/night). The young trees were then transplanted into 10 L pots filled with compost (Gramoflor Universel) and fertilized with 3 g l^−1^ of slow-release Nutricot T 100 granules (13:13:13:2 N:P:K:MgO, Fertil, Boulogne-Billancourt, France). They were further cultivated for 1 month in the same growth chamber and watered to field capacity every day. The trees retained (*n* = 48), with a view to the forthcoming O_3_ exposure experiments, were 29.5 ± 0.2 cm high, with 13.1 ± 0.1 leaves. During experiments, all foliar assessments were repeated at the third and tenth leaf position from the tree base, thus selecting the youngest fully expanded leaf and that still in expansion at treetop by the start of exposure.

Before exposure, the selected trees were randomly distributed among six ventilated phytotron chambers (1 air change min^−1^; 120 cm × 117 cm and 204 cm high) within the O_3_-exposure facility of the PEPLor platform (Faculty of Sciences and Technologies, University of Lorraine). Within each chamber (*N* = 8 trees), the plant position was randomized by each assessment. The transferred poplars were then left to acclimate for 1 week, with environmental conditions similar to those in the growth chamber. The O_3_ exposure experiment included three treatments [charcoal-filtered (CF) air; CF + 80 ppb O_3_; CF + 100 ppb O_3_] replicated in two chambers each and performed for 30 days (*N* = 16 trees per treatment). O_3_ was generated from pure oxygen using an O_3_ generator (Innovatec II, Rheinbach, Germany), and provided to the chambers during the daytime period in the form of a 13 h square wave, starting 1 h after the light was switched on. The O_3_ concentrations within each phytotron chamber were monitored twice an hour using a computer-assisted automatic O_3_ analyzer (O341M, Environment SA, Paris).

### Dynamics of Leaf Physiology Responses and Estimation of Phytotoxic O_3_ Dose

The effect of treatments on the dynamics of leaf gas exchanges was assessed by measuring the net CO_2_ assimilation rates (*A*_net_) and stomatal conductance to water vapor (*g_w_*) every 2 days, 3 h after starting the O_3_ exposure. Selecting six trees per treatment, the measurements were performed using a Li-6400XT portable photosynthesis system (LiCor, Inc., Lincoln, NE, United States), with cuvette temperature set at 22°C, light intensity (PAR) at 300 and 320 μmol m^−2^ s^−1^ for measurements at the third and tenth leaf position, respectively, airflow at 300 μmol s^−1^, CO_2_ concentration at 400 ppm, and leaf vapor pressure deficit (VPD_leaf_) < 1 kPa. The values were recorded once *g_w_* and *A*_net_ remained stable for 30 s.

The *g*_w_ estimates (mol H_2_O m^−2^ s^−1^) were used to calculate the instantaneous O_3_ uptake into the leaf under environmental stable conditions ($F_{O_{3}}$), according to Bagard et al. (2015):

$$F_{O_{3}}={\left[O_{3}\right]}_{atm}*g_{O_{3}}$$

with $F_{O_{3}}$ as the O_3_ flux (nmol O_3_ m^−2^ s^−1^), [O_3_]_atm_ as the O_3_ concentration (ppb) in the phytotron chamber, and $g_{O_{3}}$ (O_3_ m^−2^ s^−1^) as the stomatal conductance to O_3_, according to (Lamaud et al., 2009):

$$g_{O_{3}}=\frac{D_{O_{3}}}{D_{H_{2}O}}*g_{w}$$

with $D_{O_{3}}$ and $D_{H_{2}O}$ as the O_3_ and water molecular diffusivity (cm^−2^ s^−1^) respectively (Massman, 1998). The hourly O_3_ uptake (mmol O_3_ m^−2^ h^−1^) was calculated by integrating $F_{O_{3}}$ over an hour and the POD_0_ (mmol O_3_ m^−2^), by cumulating the hourly O_3_ uptake since the beginning of experiment. Missing g_w_ measurements were estimated based on values from flanking days (Bagard et al., 2008).

The effect of treatments on the dynamics of leaf chlorophyll content was assessed by measuring estimates of surface-based concentrations of chlorophylls (total chlorophyll index) every day, 1 h after switching the light on and before the start of O_3_ treatment. Selecting six trees per treatment, the estimates were obtained averaging 10 measurements per leaf performed with a leaf clamp sensor device (Dualex Force-A, Orsay, France).

### Dynamics of Microscopic and Visible Leaf Injury

The development of HR-like lesions within the mesophyll and that of O_3_ symptoms throughout foliage was monitored using completing microscopic assessments and visible injury observations. For microscopic assessments, two discs (diameter = 6 mm) per leaf position in two trees per chamber were sampled every 2 days, until HR-like lesions were detected in the 100 ppb O_3_ treatment at both leaf positions; the sampling interval was then extended (3–6 days). The harvested discs were processed immediately after sampling.

Necrotic cells within mesophyll as a consequence of HR-like lesions were evidenced using the Trypan blue assay (Pasqualini et al., 2003; Joo et al., 2005; Faoro and Iriti, 2009). Briefly, the leaf discs were stained for 3 min in a hot lactophenol Trypan blue mixture (60 ml staining solution: 10 g phenol, 10 mg Trypan blue, 30 ml ethanol, 10 ml glycerol, 10 ml lactic acid, and 10 ml distilled water) and the necrotic cells contrasted for 20 min against a clear background using 2.5 g ml^−1^ hot chloral hydrate, before mounting in 60% glycerol (Pasqualini et al., 2003). The preparations were then transferred to WSL where all microscopy assessments were performed. The disk’s central part, free of staining artifacts, was observed using the 5× objective of a Leica microscope (Leitz DM/RB). Given the disk thickness (>200 μm) and to create high contrast pictures, the preparations were imaged after inserting the 10x condenser and removing most filters and diaphragms, using the INFINITY 2-1R camera and Lumenera Infinity Analyze (release 6.4) software (Lumenera Corp., Ottawa, ON, Canada). The center of each disk preparation was photographed, creating composite images made of nine tiles each. The percentage area, particle size, and shape properties of HR-like lesions inside of stitched images were quantified using computer-assisted color image analysis (software WinCELL™ 2004, Regent Instruments Inc., Québec, QC, Canada). Briefly, the software attributed the whole lesion or part of it to one of two color classes (non-oxidized: violet hue; oxidized: dark blue hue) made of 10 shades each, defined based on a representative batch of images and contrasting with the background color class (grayish hue, based on 10 white to gray shades). The quantified parameters characterized the size and shape properties of total and individual lesion particles.

The HR-like lesions and oxidation diagnosis were verified based on hand, and semi-thin sections from samples collected in all treatments at the two leaf positions during the whole study and subsequently processed and observed as described previously (Moura et al., 2018). Briefly, supplementary leaf discs were infiltrated upon sampling with EM-grade 2.5% glutaraldehyde buffered at pH 7.0 with 0.067 M Soerensen phosphate buffer, renewed after vacuum infiltration. Sections (60 μm) obtained using a custom-made hand microtome and kept unstained were used for visualizing chlorophylls and the oxidation of HR-like lesions. Technovit-embedded 1.5 μm sections, obtained using a Supercut Reichert 2050 microtome and stained with Toluidine blue (Vollenweider et al., 2016), were used to identify HR-like markers within necrotic mesophyll cells, after observation with phase contrast illumination in bright field microscopy using the 5–100× objectives of the Leica microscope and imaged using the Infinity camera, as mentioned above.

The development of O_3_ injury in response to treatments was surveyed in the morning, before the start of O_3_ exposure, daily. Upon appearance, the development of visible injury was monitored with pictures of symptomatic leaves. The percentage area of necrosis per leaf within the latter material was quantified using color image analysis, using the Color Segmentation plugin in Fiji freeware (ver. 2.0.0; Schindelin et al., 2012).

### Morphological Assessments

After 30 days of exposure, all trees were harvested and biometric assessments were conducted. Tree height was recorded and stem diameter 1.5 cm above root collar was measured, using a hand caliper. The number of leaves per tree, shed or still attached, was recorded before harvest. Leaf and stem material was oven-dried to constant weight, before determining the dry mass of each fraction.

### Statistical Analysis

The dynamics of physiological and structural responses to treatments in foliage and the differences in whole-tree morphology and biomass between groups by the end of the experiment were analyzed using linear mixed-effects models (lmem). The fixed-effect factors included the O_3_ treatment, leaf position, time or POD_0_ and interactions, whereas the tree nested in the chamber (leaf data; with the leaf position as the statistical unit) or the chamber (morphology/biomass data; with the tree as the statistical unit) were introduced in models as random terms. Homoscedasticity and normality of residuals were verified graphically, and the dependent variables were log- or square-transformed to meet the model assumptions, as needed. The differences between treatments at given assessment dates were tested using *post-hoc* tests (Tukey’s honest significant difference). All statistical analyses were performed using R statistical software, version 3.5.0 (R Development Core Team, 2017), with the packages lme4 (Bates et al., 2015) for linear mixed-effects models, and emmeans (Lenth, 2016) for *post-hoc* testing.

## Results

### Morphological Responses

After 30 days of exposure, no change in tree height, stem diameter, or foliar dry mass in response to O_3_ exposure was observed (Table 1). However, the stem biomass (*p* = 0.014), amount of leaves (*p* = 0.003), and leaf shedding (*p* = 1.2 × 10^−6^) were increased, with significant differences between the 80 and 100 ppb O_3_ treatments in the case of leaf shedding.

**Table 1:** Morphological responses to O_3_ treatments in hybrid poplar (*Populus tremula x alba*) at the end of the experiment.

Treatment	Tree height (cm)	Stem diameter (mm)	Foliage biomass (g)	Stem dry mass (g)	Leaf shedding (%)	Leaf number
Charcoal-filtered	100.31 ± 0.95	9.93 ± 0.18	30.09 ± 0.86	15.72 ± 0.53a	1.59 ± 0.81a	33.31 ± 0.54a
80 ppb ozone	104.38 ± 1.66	10.16 ± 0.33	31.54 ± 1.50	17.51 ± 0.91b	7.41 ± 1.99b	35.00 ± 0.61b
100 ppb ozone	101.75 ± 0.81	10.23 ± 0.12	28.83 ± 0.61	16.50 ± 0.39ab	16.94 ± 2.21c	35.44 ± 0.39b
Treatment	ns	ns	ns	∗	∗∗∗	∗∗

model: lmer(variable) ~ O_3_ treatment + (1 | chamber); ∗∗∗*p* ≤ 0.001; ∗∗*p* ≤ 0.01; ∗*p* ≤ 0.05; ns, not significantly different. Values represent means ± SE, *n* = 16. Different letters indicate significant differences between treatments by the end of experiment (*post-hoc* Tukey’s honest significant difference, *p* ≤ 0.05).

### Dynamics of Stomatal Responses and Changes in the Phytotoxic O_3_ Dose

**Figure 1:**
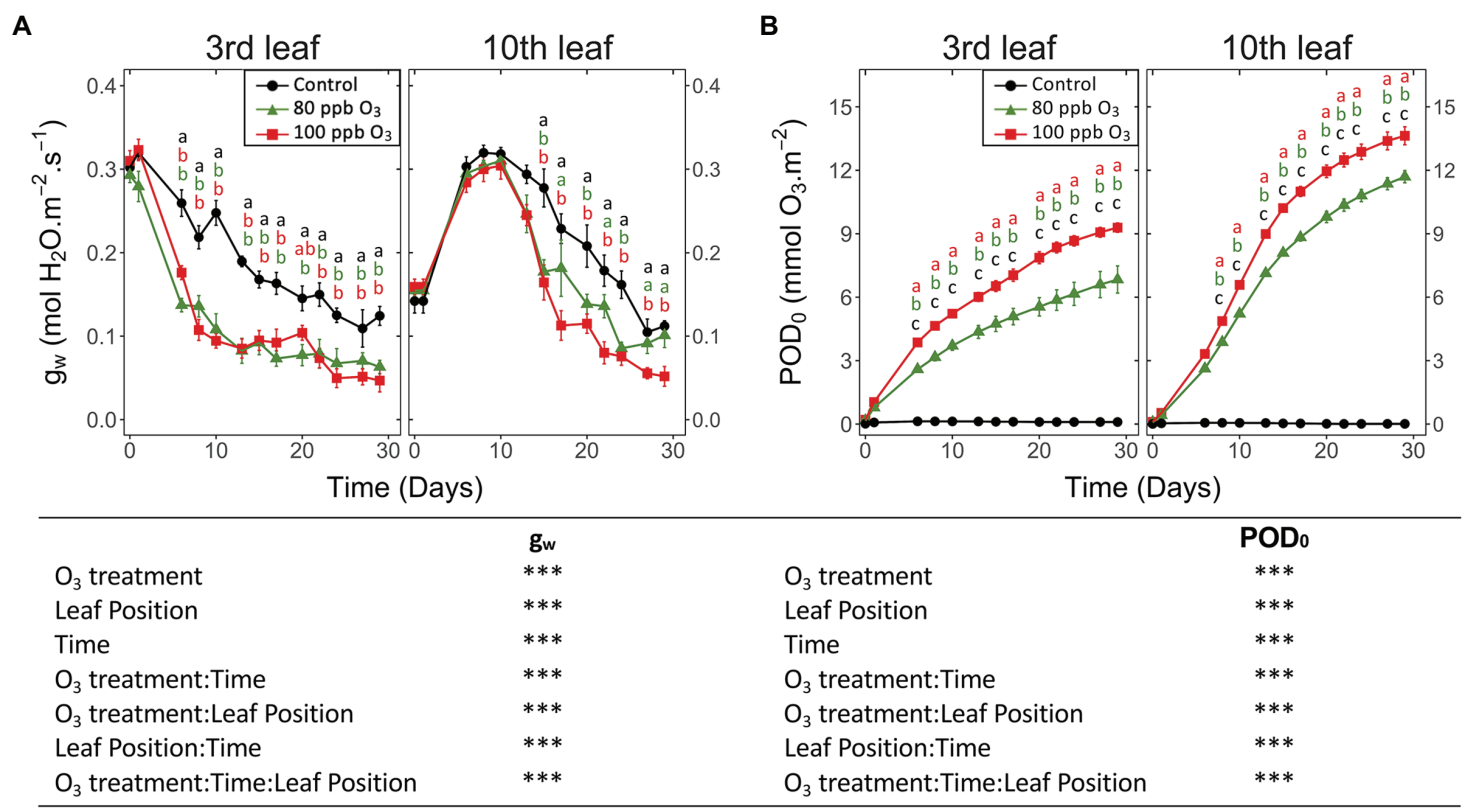
Dynamics of changes in the **(A)** stomatal conductance to water (g_w_) and **(B)** phytotoxic O_3_ dose (POD_0_) of hybrid poplar leaves (*Populus tremula x alba*), as a function of O_3_ treatment (charcoal-filtered air ●, 80 ppb O_3_ ▲, 100 ppb O_3_ ◼), leaf position, time of assessment and interactions {model: lmer[sqrt(variable)] ~ O_3_ treatment ∗ leaf position ∗ time + (1 | tree / chamber); ∗∗∗ *p* ≤ 0.001}. Values represent means ± SE, *n* = 6. Different letters indicate significant differences between treatments for a given assessment date (*post-hoc* Tukey’s Honest significant difference, *p* ≤ 0.05).

At both leaf positions, the 100 and 80 ppb O_3_ treatments significantly reduced *g_w_* (Figure 1A; O_3_ treatment: *p* < 0.001) and accelerated its leaf ontology-driven decrease (O_3_ treatment∗Time: *p* < 0.001). This reduction was delayed at the tenth vs. third leaf position (O_3_ treatment*leaf position, O_3_ treatment∗time∗leaf position: *p* < 0.001), with a 50% decrease in *g_w_* reached in 15 vs. 6 days, respectively, in the 100 ppb treatment. As indicated by increasing *g_w_* in maturing leaves (tenth leaf position) during the first 10 days of exposure irrespective of O_3_ exposure (leaf position: *p* < 0.001), the O_3_ treatment affected *g_w_* only once the ontological development had been achieved (latency phase, Figure 1A). By the end of the experiment and only at the tenth leaf position, the differences in *g_w_* between the 100 and 80 ppb treatments were significant.

By the end of the experiment, trees in the 100 vs. 80 ppb O_3_ treatment showed a larger POD_0_, as a consequence of their higher O_3_ concentrations and mostly similar g_w_ (Figure 1B; O_3_ treatment, O_3_ treatment*time: *p* < 0.001). After 30 days of exposure, the POD_0_ at the third and tenth leaf positions was thus 1.4 and 1.2 times higher in the 100 vs., 80 ppb O_3_ treatments. The POD_0_ was also higher in leaves at the tenth vs. third leaf position (leaf position: *p* < 0.001), as a consequence of the delayed leaf ontogeny and higher g_w_ (O_3_ treatment*leaf position: *p* < 0.001). After 10 days of exposure, the POD_0_ levels in younger foliage thus exceeded those in older material by approximately 25% and outpaced them by 40% by the end of exposure (O_3_ treatment∗time∗leaf position: *p* < 0.001).

### Dynamics of Leaf Physiology Responses

**Figure 2:**
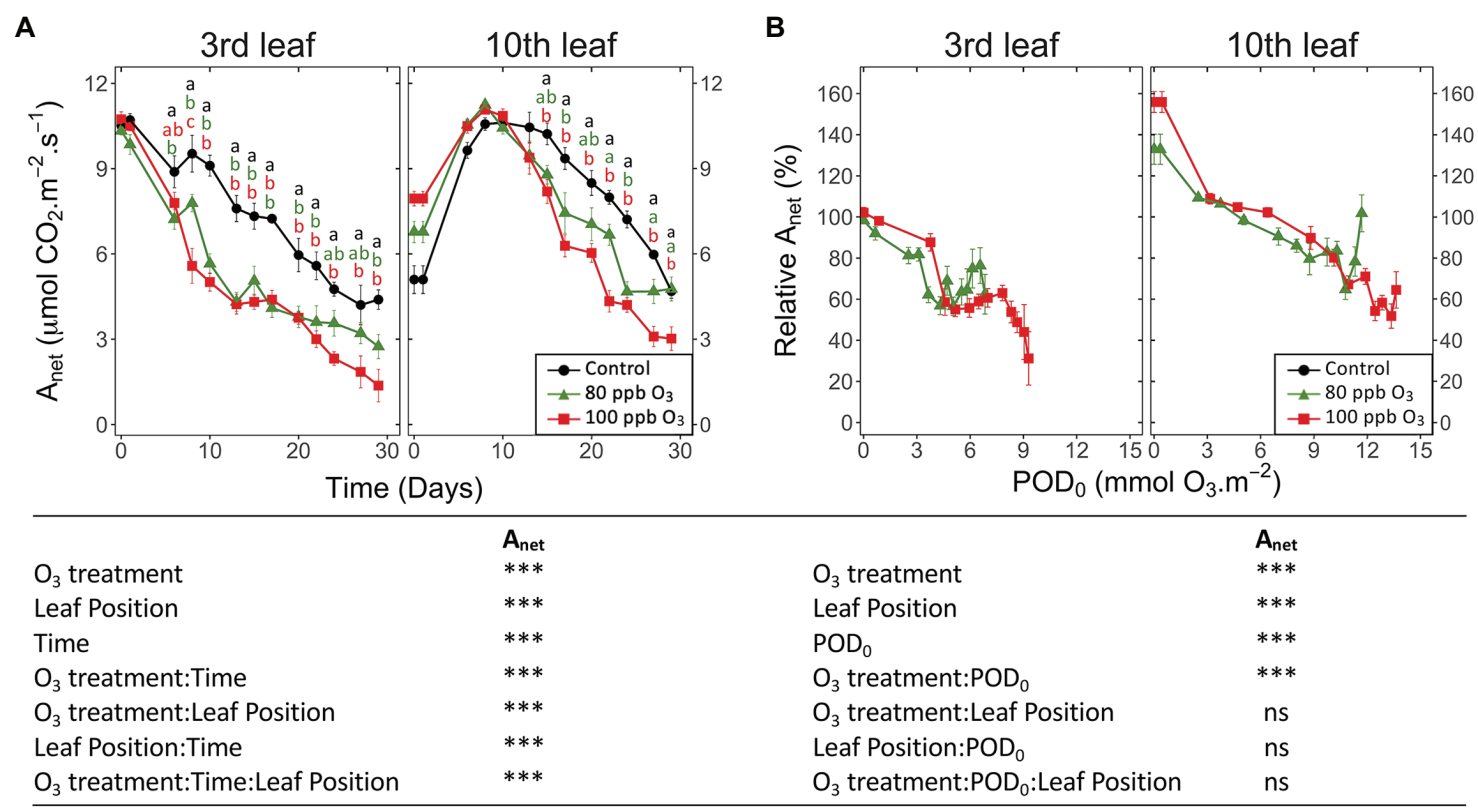
Dynamics of changes in the net CO_2_ assimilation (A_net_) of hybrid poplar leaves (*Populus tremula x alba*), as a function of assessment time **(A)**, phytotoxic O_3_ dose (POD_0_; **B**), O_3_ treatment (charcoal-filtered air ●, 80 ppb O_3_ ▲, 100 ppb O_3_ ◼), leaf position and interactions [model: lmer(variable) ~ O_3_ treatment ∗ leaf position ∗ time + (1 | tree / chamber); ∗∗∗ *p* ≤ 0.001; ns, not significantly different]. Values represent means ± SE, *n* = 6. Different letters indicate significant differences between treatments at a given assessment date (*post-hoc* Tukey’s Honest significant difference, *p* ≤ 0.05).

Irrespective of the leaf position, *A*_net_ showed responses to treatments and response dynamics similar to *g*_w_ (Figure 2A vs. Figure 1A). After 30 days of exposure, *A*_net_ in the 100 ppb O_3_ vs. CF treatment was decreased by 70 and 35% at the third and tenth leaf position, respectively. Once the leaf ontogenetic differentiation achieved, *A*_net_ decreased with POD_0_ in a constant and monotonic manner (Figure 2B; POD: *p* < 0.001), regardless of the treatment or leaf position. The significant effects of O_3_ treatment and O_3_ treatment∗POD factors (*p* < 0.001) could then be related to the less affected A_net_ values in the 80 vs. 100 ppb treatment at the highest POD_0_ reached by the end of the exposure. The observed reduction in A_net_ as a function of POD_0_ was stronger at the third vs. tenth leaf position (Figure 2B; leaf position *p* < 0.001) but the dynamics at both leaf positions was similar (leaf position∗POD: ns). Hence, at POD_0_ of 5 mmol O_3_ m^−2^, *A*_net_ at the tenth leaf position did not show any reduction relative to CF treatment yet, vs. 50% *A*_net_ loss in older leaves, irrespective of the O_3_ treatment. The response differences between the two leaf positions were further observed at higher POD_0_. With POD_0_ above 9 mmol O_3_ m^−2^, as recorded in younger leaves only, and also as a consequence of the aforementioned latency effect (Figure 1B), A_net_ never dropped to levels observed at the third leaf position for lower POD_0_. Consequently, the photosynthetic activity in younger vs. older foliage appeared less sensitive to the absorbed O_3_ dose.

**Figure 3:**
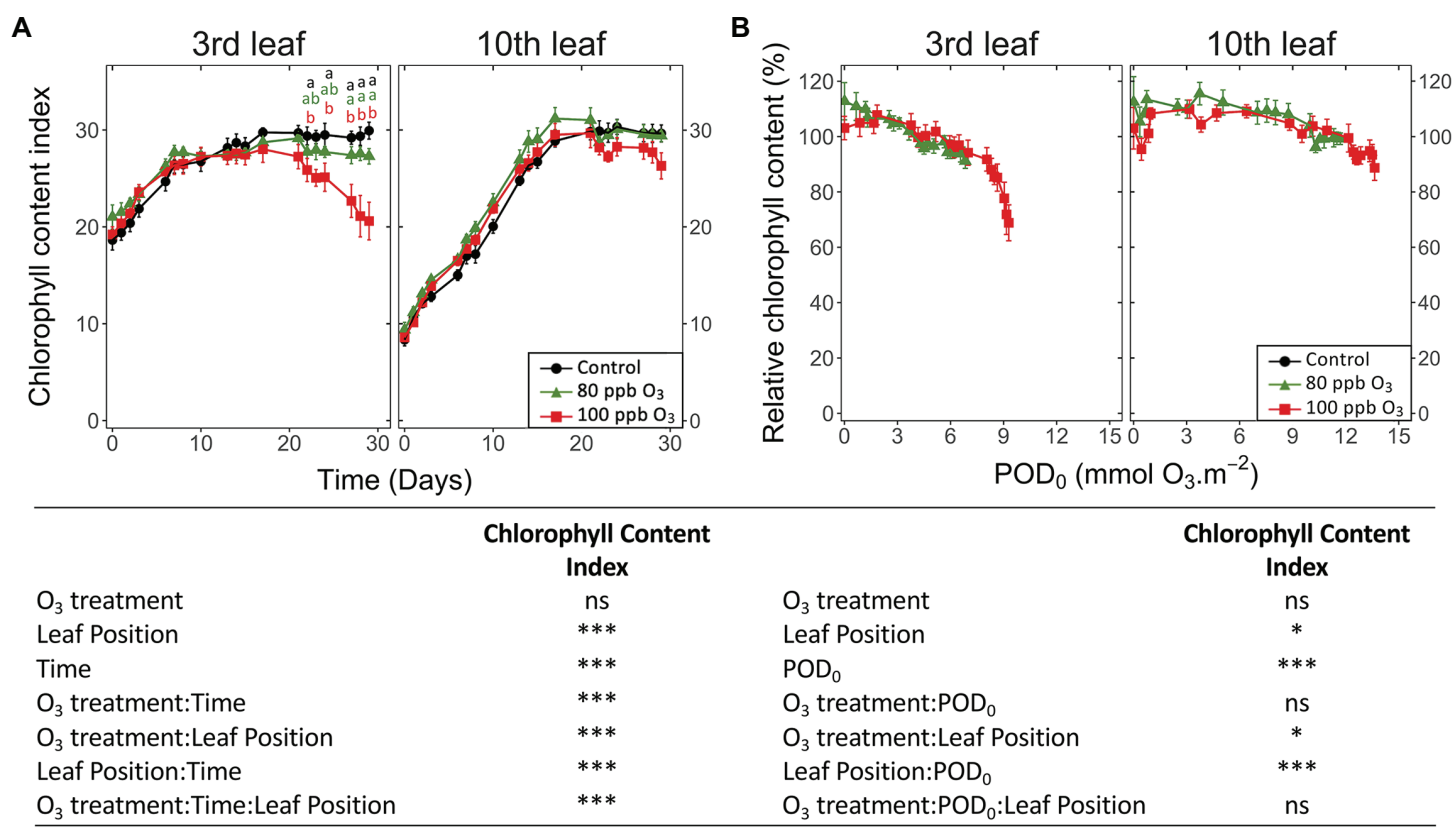
Dynamics of changes in the surface-based concentration of chlorophylls (total chlorophyll content index of Dualex) within hybrid poplar leaves (*Populus tremula x alba*), as a function of assessment time **(A)**, phytotoxic O_3_ dose (POD_0_; **B**), O_3_ treatment (charcoal-filtered air ●, 80 ppb O_3_ ▲, 100 ppb O_3_ ◼), leaf position and interactions [model: lmer(variable) ~ O_3_ treatment ∗ leaf position ∗ time + (1 | tree / chamber); ∗∗∗ *p* ≤ 0.001; ∗ *p* ≤ 0.05; ns, not significantly different]. Values represent means ± SE, *n* = 6. Different letters indicate significant differences between treatments at a given assessment date (*post-hoc* Tukey’s Honest significant difference, *p* ≤ 0.05).

An O_3_ impact on the chlorophyll content index of leaves was detected after 24 days of the experiment. The impact was restricted to the third leaf position (Figure 3A; O_3_ treatment: ns; O_3_ treatment∗time, O_3_ treatment*leaf position: *p* < 0.001), showing a decrease of 30% for the total chlorophyll index in the 100 ppb O_3_ vs. CF treatment. These findings primarily related to latency effects due to leaf ontological maturation, which were observed at the third as well as the tenth leaf position in the case of this parameter, lasting 7 and 17 days, respectively. Accordingly, a larger latency peak was observed in younger than older foliage. The total chlorophyll index decreased with higher POD_0_ (Figure 3B; POD: *p* < 0.05), irrespective of the O_3_ treatment (O_3_ treatment: ns, O_3_ treatment∗POD: ns). At low POD_0_, the decline was rather monotonic, but accelerated with values exceeding 8 mmol m^−2^ at the third leaf position, thus contrasting with the nearly linear drop observed in younger leaves. Confirming a higher O_3_ tolerance leaves at the tenth leaf position showed smaller (leaf position: *p* < 0.05) and slower (leaf position∗POD_0_ *p* < 0.001) drops with higher POD_0_.

### Dynamics of HR-Like Lesion Spread and Development

**Figure 4:**
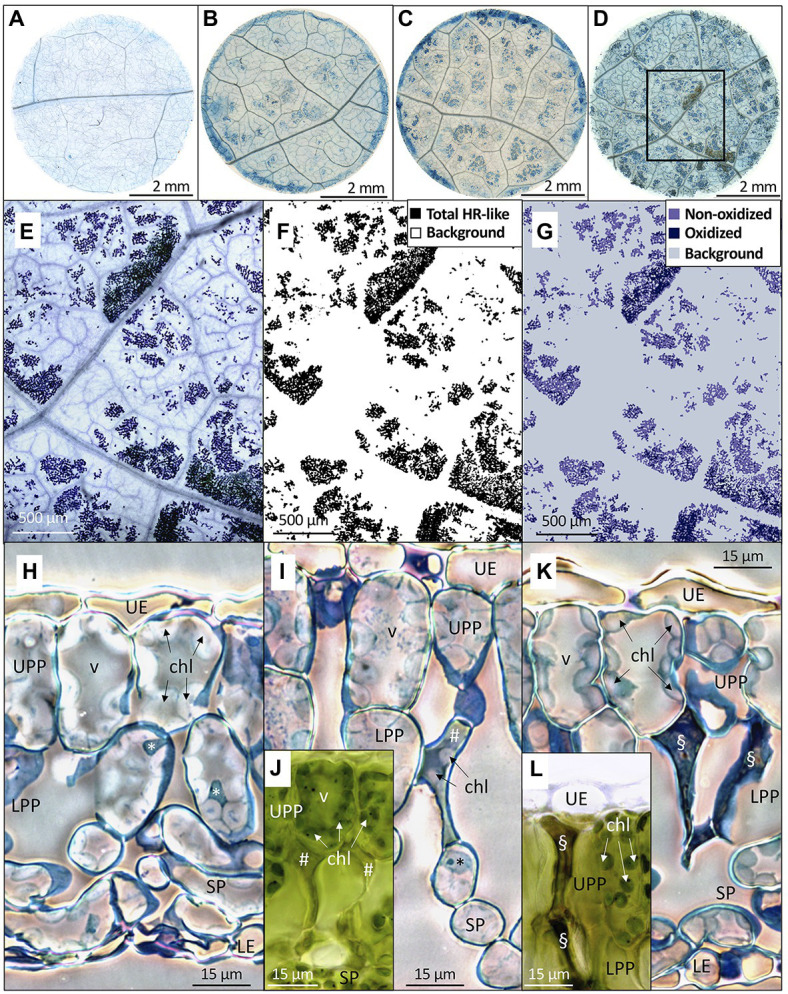
Distribution, morphology, and structural properties of HR-like lesions within hybrid poplar leaves (*Populus tremula x alba*). **(A–D)** Foliar discs excised at low leaf position (third) from leaves exposed to 100 ppb O_3_ during 2 **(A)**, 8 **(B)**, 13 **(C)**, and 27 **(D)** days. **(A)** Central area within each disk was photographed each time (frame in **D**). **(E–G)** Image analysis of HR-like lesion after 27 days of treatment, as framed in **(D)**. **(E)** Synthetic digital image, made of 9 stitched micrographs (5x magnification). The lesions are separated by veinlets and non-symptomatic tissues. **(F)** The binary image of total HR-like lesion vs. background (white). **(G)** Classification of HR-like injuries into oxidized and non-oxidized lesion groups, based on color classes. **(H–L)** Changes in the mesophyll tissue and cell structure underlying the HR-like lesions. **(H)** Asymptomatic leaf tissues in a leaf sample from the filtered air treatment. I–L: necrotic cells within the upper (UPP) and lower (LPP) palisade parenchyma underlying the HR-like lesions. **(I,J)** Within mesophyll cells having recently undergone HR-like necrosis (#), the chloroplasts (chl) were still visible and had retained their green color **(J)**. **(K,L)** at a later stage, the HR-like lesions (§) showed cell-content disruption and oxidation **(L)**. Other structures: UE, upper epidermis; SP, spongy parenchyma; LE, lower epidermis; v, vacuole; *, nucleus. Technical specifications: staining with Trypan blue **(A–E)** and Toluidine blue **(H,I,K)**; observations in bright field microscopy **(A–E,H–L)** using phase-contrast **(H,I,K)**; **(J,L)** fresh, unfixed and unstained leaf sample preparations.

**Figure 5:**
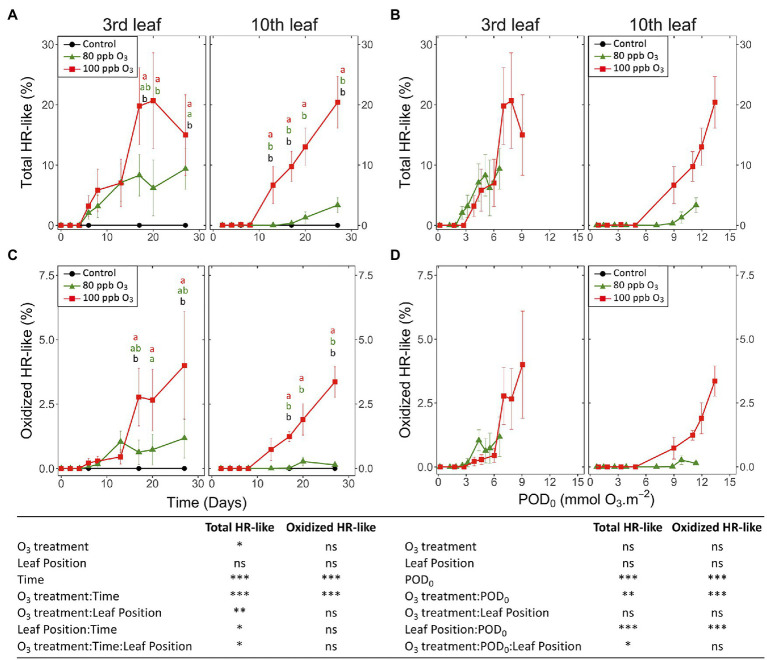
Development dynamics of HR-like lesions **(A,B)** and lesion oxidation **(C,D)** in hybrid poplar leaves (*Populus tremula x alba*), as a function of the assessment time **(A,C)**, phytotoxic O_3_ dose (POD_0_; **B,D**), O_3_ treatment (charcoal-filtered air ●, 80 ppb O_3_ ▲, 100 ppb O_3_ ◼), leaf position and interactions {model: lmer[sqrt(variable)] ~ O_3_ treatment ∗ leaf position ∗ time + (1 | tree / chamber); ∗∗∗*p* ≤ 0.001; ∗∗*p* ≤ 0.01; ∗*p* ≤ 0.05; ns, not significantly different}. Values represent means ± SE of percentage area of leaf discs showing microscopic injury, *n* = 4. Different letters indicate significant differences between treatments at a given assessment date (*post-hoc* Tukey’s honest significant difference, *p* ≤ 0.05).

**Figure 6:**
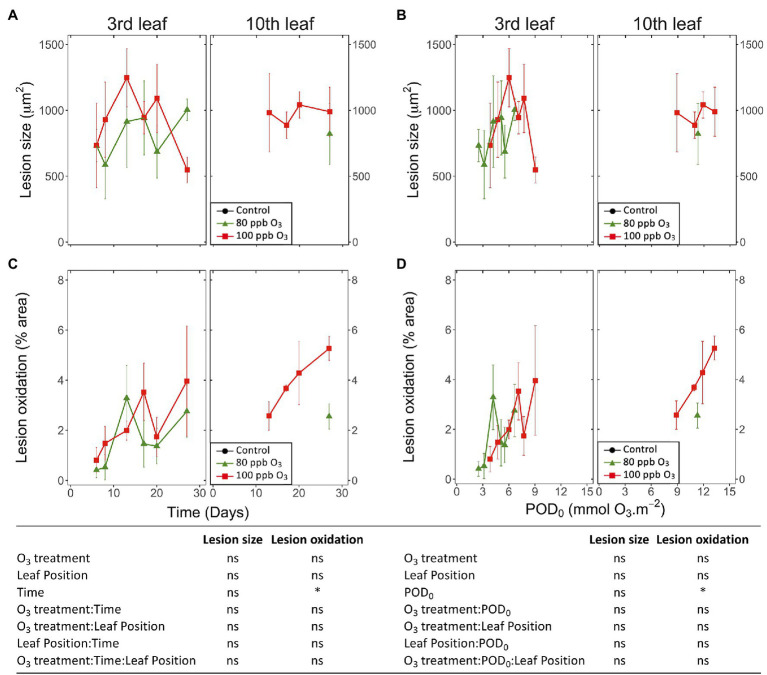
Dynamics of changes in the size **(A,B)** and degree of oxidation **(C,D)** of single HR-like lesions in hybrid poplar leaves (*Populus tremula x alba*), as a function of the assessment time **(A,C)**, phytotoxic O_3_ dose (POD_0_; **B,D**), O_3_ treatment (charcoal-filtered air ●, 80 ppb O_3_ ▲, 100 ppb O_3_ ◼), leaf position and interactions [model: lmer(variable) ~ O_3_ treatment ∗ leaf position ∗ time + (1 | tree/chamber); ∗ *p* ≤ 0.05; ns, not significantly different, na not tested]. **(A,B)** The size of HR-like lesions did not respond to the experimental factors and interactions. Values represent means ± SE of distinct lesion size **(A,B)** and percentage area showing oxidation **(C,D)**, *n* = 4.

The microscopic necrosis observed in mesophyll using the Trypan blue assay was diagnosed as being caused by HR-like processes (Figures 4A–D), based on several typical O_3_-stress markers (Paoletti et al., 2009; Vollenweider et al., 2019). They included (1) the characteristic intercostal distribution of lesions (Figure 4E), (2) the development of injury first in older leaves (Figure 5A), or (3) the multiple HR-like events restricted to cells or small groups of cells within mesophyll (Figures 4E–G,I vs. Figure 4H). Collapsed dead cells were mainly observed in the lower palisade parenchyma (Figures 4I–L). Non-oxidized lesions (Figure 4G) showed up first (Figures 5A,C, 6C), with, for instance, still green chloroplasts visible within collapsed dead cells (Figures 4I,J). The dark hues of oxidized lesions (Figure 4L) were enhanced by staining with Trypan blue (Figures 4E,G). Oxidized cells showed sharp wall angles indicative of breaks and disrupted cell content (Figures 4K,L). All these typical HR-like traits showed little variation, regardless of the O_3_ treatment or leaf position.

The first HR-like lesions at the third and tenth leaf position were observed after 6 and 13 days of exposure, respectively. This was much earlier than reductions in the chlorophyll content index (Figure 5A). Despite large response variability among trees, the effect of O_3_ treatment was significant (Figure 5A; O_3_ treatment: *p* < 0.05; O_3_ treatment*time: *p* < 0.001). A larger leaf percentage area showing HR-like lesions was observed for the 100 vs. 80 ppb O_3_ treatment, with differences between the two treatments at the third leaf position becoming significant after 20 days of exposure (O_3_*time; *p* < 0.001). After 27 days of treatment, the percentage area of lesions in the 100 vs. 80 ppb O_3_ treatment was two and five times higher at the third and tenth leaf positions, respectively. However, each leaf position showed specific response dynamics (O_3_ treatment∗leaf position: *p* < 0.01, O_3_ treatment∗time∗leaf position: *p* < 0.05), rather sigmoidal-like vs. linear – once lesions appeared – in older vs. younger foliage (Figure 5A). Moreover, the HR-like lesions in response to the two O_3_ concentrations showed up simultaneously at the third leaf position whereas a 7-day delay was observed at the tenth leaf position (Figure 5A).

When expressed as a function of POD_0_, the differences between the two O_3_ treatments in the leaf percentage area showing HR-like lesions were leveled out, especially at the third leaf position (Figure 5B; O_3_ treatment: ns). However, the dependency on POD_0_ was lessened at the tenth vs. third leaf position (leaf position∗POD: *p* < 0.001), and distinctly higher lesion percentage areas in response to similar POD_0_ were observed in the 100 vs. 80 ppb O_3_ treatment in younger leaves (O_3_ treatment∗POD∗leaf position: *p* < 0.05). Hence, not only the O_3_ dose but also the O_3_ absorption rate then determined the lesion severity. The oxidized HR-like lesions, expressed as a function of time or POD_0_, showed responses and response dynamics similar to HR-like lesions taken globally (Figures 5C,D vs. Figures 5A,B). The main differences included a smaller percentage of the injured area and a weaker symptom dynamics. At the tenth leaf position, oxidized HR-like lesions in the 80 ppb O_3_ treatment were observed only occasionally.

Analyzing single HR-like injuries, the shape (data not shown) and size of lesions remained stable over time or with increasing POD_0_. Furthermore, they did not respond to the O_3_ treatment, leaf position, or interaction factors (Figures 6A,B; all factors: ns). The only change observed was increasing oxidation with longer exposures and at higher POD_0_ (Figures 6C,D; Time, POD: *p* < 0.05). Hence, the observed increases in the percentage area of HR-like lesions with time, or higher POD_0_ and in response to the O_3_ treatment (Figures 5A,B) resulted as a consequence of the multiplication of single HR-like reactions and higher lesion density, rather than from increased growth of already developed injuries. However, both the higher lesion density and the growing oxidation of individual HR-like lesions could contribute to the observed increase in the leaf percentage area showing oxidation (Figures 5C,D).

### Emergence of Visible Symptoms

**Figure 7:**
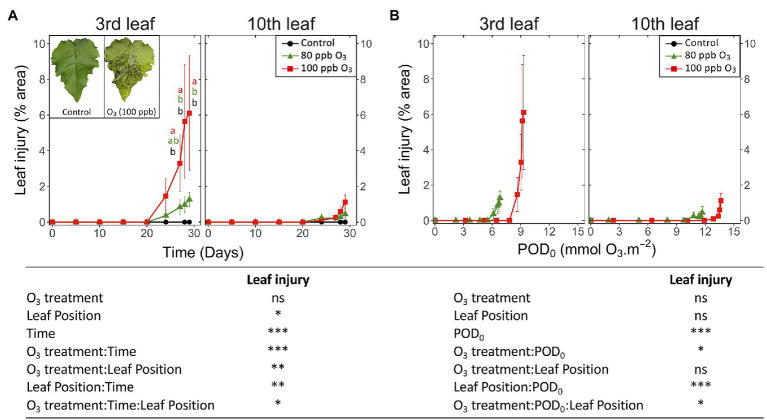
Development dynamics of visible O_3_ injury in hybrid poplar leaves (*Populus tremula x alba*), as a function of the assessment time **(A)**, phytotoxic O_3_ dose (POD_0_; **B**), O_3_ treatment (charcoal-filtered air ●, 80 ppb O_3_ ▲, 100 ppb O_3_ ◼), leaf position and interactions {lmer[log(variable)] ~ O_3_ treatment ∗ leaf position ∗ time + (1 | tree / chamber); ∗∗∗*p* ≤ 0.001; ∗∗*p* ≤ 0.01; ∗*p* ≤ 0.05; ns, not significantly different}. The insert in **(A)** shows typical O_3_ symptoms, in the form of intercostal necrotic dark spots, within a leaf at third leaf position exposed for 30 days (POD_0_ = 9.3 mmol O_3_ m^−2^, 100 ppb O_3_ treatment) vs. an asymptomatic leaf from the CF treatment, sampled at the same leaf position and the end of treatment. Values represent means ± SE of leaf percentage area showing visible injury, *n* = 6. Letters indicate significant differences between treatments at a given assessment date (*post-hoc* Tukey’s Honest significant difference, *p* ≤ 0.05).

The first visible symptoms in leaves exposed to the O_3_ treatments appeared by the end of the experiment, that is, 23 days after the start of exposure. The first HR-like lesions had been detected more than 2 weeks earlier, whereas the observed drops in foliar chlorophyll content index were rather synchronous (Figure 7A vs. Figures 3A, 5A). These visible symptoms consisted of intercostal necrotic dark spots spread in the leaf blade, as previously observed in poplar (Figure 7A; Cabane et al., 2004; Giacomo et al., 2010; Dghim et al., 2013). By the end of exposure, only low levels of injury could develop (O_3_ treatment: ns; O_3_ treatment*Time *p* < 0.001), with significantly higher percentages in the 100 vs. 80 ppb O_3_ treatment at the third leaf position only. In younger leaves, the visible injury appeared 4 days later than at the third leaf position, and differences between treatments throughout the experiment remained non-significant (leaf position, O_3_ treatment∗time∗leaf position: *p* < 0.05; O_3_ treatment*leaf position: *p* < 0.01).

When expressed as a function of POD_0_, the visible injuries appeared at a lower O_3_ threshold at the third than tenth leaf position (9 and 12 mmol O_3_ m^−2^ in the case of 100 ppb treatment; Figure 7B). With only the beginning of injury development assessed in a 30-day experiment, the O_3_ treatment effects could not reach any significance, and only preliminary information on the dynamics of visible symptom development was thus obtained. The simultaneous detection of early injury in the two O_3_ treatments (Figure 7A) thereby resulted in higher injury values for similar POD_0_ in the 80 vs. 100 ppb O_3_ treatment (Figure 7B; O_3_ treatment∗POD: *p* < 0.05). Similarly, differences between younger and older leaves were detected as a trend only, with still very low injury values recorded at the tenth leaf position and for the higher POD_0_ values only (leaf position: ns; leaf position∗POD: *p* < 0.001).

## Discussion

### Dynamics of Physiological and Structural Responses to O_3_ Stress

The physiological and structural responses detected during 30 days of O_3_ exposure developed mostly before and in some cases at the same time as the initial visible injury and first leaf shedding, whereas no O_3_ effect on the gross morphology of trees was observed. The two O_3_ treatments accelerated the ontological decline of leaf gas exchange at the two-leaf positions, once leaf physiology had reached maturity. O_3_-induced reductions in the stomatal conductance and net CO_2_ assimilation are well-documented in the case of various species, including poplars (Pell et al., 1992; Bagard et al., 2015; Dusart et al., 2019a) but more rarely with a leaf ontogenetic perspective. The decrease in net CO_2_ assimilation could result from a smaller stomatal aperture, limiting CO_2_ availability. Indeed, the O_3_ effects on stomata are well-established (Kangasjarvi et al., 2005), including slower movements of cell guard cells upon exposure referred to as stomatal sluggishness, as observed in different species but with higher measurement frequency than in our case (Paoletti and Grulke, 2010; Dumont et al., 2013; Dusart et al., 2019b). Lower mesophyll conductance could also contribute to the observed acceleration of *A*_net_ reduction, as compared with the ontogenetic decrease observed in CF trees (Xu et al., 2019). Although not significant in older foliage, the drop in *A*_net_ tended to be more expressed in the 100 vs. 80 ppb treatment after 20 days, suggesting an additional reduction in carboxylation efficiency and rate of electron transport (Bagard et al., 2008; Shang et al., 2017). A possible cause could be the starting degradation of the photosynthetic machinery, as suggested by the concomitant reduction in the chlorophyll content index.

The reduction in leaf chlorophyll content is another well-documented leaf physiological response to elevated O_3_ (Reich, 1983; Bagard et al., 2015; Dusart et al., 2019b), indicative of ACS, together with other markers of chloroplast degeneration (Mikkelsen and HeideJorgensen, 1996; Günthardt-Goerg and Vollenweider, 2007; Moura et al., 2018). Also typical of ACS and degenerative processes was the progressive and mostly monotonic reductions observed in the case of all physiology parameters (*g_w_*, *A*_net_, and chlorophyll index). This contrasted with abrupt responses upon exceedance of a threshold, as observed in the case of HR-like reactions. In the field, the ontogeny-driven ACS latency observed in both younger and older foliage (total chlorophyll index) may also contribute to delaying the onset of degenerative events in response to O_3_-stress, as suggested by the development of ACS traits and visible injury rarely occurring before summer, once the foliage has fully matured (Vollenweider et al., 2019).

The new cell necrosis assay, using computer-assisted color image analysis, allowed us to monitor the emergence and development of validated HR-like reactions for the first time. It provided unprecedented capacity for quantitative assessments of cell death reactions in experimental conditions, overcoming limitations and uncertainties regarding the observation of visible injury only. Typical HR-like markers were detected in the lesions (Paoletti et al., 2009; Vollenweider et al., 2013, 2019; Feng et al., 2016). However, there were marked differences in classical traits as well, including the mid- rather than the upper-mesophyll location of HR-like lesions or a missing intra- and intercellular gradient of injury. Such features indicated low levels of photo-oxidative stress (Foyer et al., 1994; Günthardt-Goerg and Vollenweider, 2007; Guerrero et al., 2013). Given maximum PAR above 2000 vs. 350 μmol m−2 s^−1^ with high (Ritchie, 2010; Poorter et al., 2019) vs. low light conditions, HR-like reaction peculiarities – together with the late onset of ACS – can be attributed to specifics in the environmental conditions, especially regarding PAR supply. This finding thus provides further confirmation of the close dependency relating the O_3_ symptom expression in foliage and precise experimental and exposure conditions of tested material (Paoletti et al., 2009; Moura et al., 2018; Vollenweider et al., 2019).

The rather sigmoidal-like injury dynamics observed in older foliage was in good agreement with already existing molecular and trait evidence on HR-like processes. The 6 days/3 mmol O_3_ m^−2^ s^−1^ delay between the start of exposure and occurrence of first lesions was thus indicative of the O_3_ dose-dependent onset of genetically controlled PCD (Rao et al., 2000; Overmyer et al., 2005). The steep increase in injury percentage area reflected the rapid cell death completion once PCD started (Overmyer et al., 2005; Günthardt-Goerg and Vollenweider, 2007). Finally, the plateau reached was indicative of lesion containment, blocking its further spread (Overmyer et al., 2003; Kangasjarvi et al., 2005; Marchica et al., 2019). In younger foliage, the experiment was terminated before a plateau could be reached, with values of lesion percentage area which would probably have been sizably higher than at the third leaf position. Further suggesting the genetic control of PCD, in older foliage, the first HR-like reactions occurred 17 days/at 5.3 mmol O_3_ m^−2^ s^−1^ before any evidence of biochemical limitation and chloroplast injury, as indicated by low levels of chlorophyll content indexes. The early HR-like responses, and their antecedence concerning ACS and first visible injury, contrasted with field evidence (Vollenweider et al., 2019), further outlining how important the environmental conditions can be regarding response order and dynamics.

Image analysis in WinCELL based on three color classes allowed us to quantify the structurally contrasted non-oxidized and oxidized lesions, based on constitutive and stained-color characteristics. REDOX changes during oxidative stress and cell death form an important cell physiology process (Foyer and Noctor, 2005) that is well-documented in the case of O_3_ stress (Ranieri et al., 2000; Baier et al., 2005; Chen and Gallie, 2005; Bellini and De Tullio, 2019), detectable with different structural and ultrastructural markers (Moura et al., 2018; Vollenweider et al., 2019) and underlying changes in visible symptom expression. Expressed as leaf area percentages, the oxidized and total HR-like lesions showed similar dynamics and responses to increasing POD_0_ (same results for non-oxidized lesions, data not shown). The main differences in oxidized vs. non-oxidized lesions included their (1) lower percentage area, (2) delay in development, and (3) higher severity (i.e., cell wall breaks, cell content disruption). Non-oxidized and oxidized lesions may thus correspond to two types or two stages of HR-like reactions. However, the first hypothesis appears unlikely, given lacking molecular evidence for alleged PCD severity variation. In favor of the second, oxidized vs. non-oxidized lesions appeared later, and the oxidation degree of a lesion increased with time. However, it implies the further evolution of HR-like lesions after cell collapse and death, which needs further structural and ultrastructural confirmation.

The visible injury was detected only once 2–5% of the leaf percentage area showed oxidized lesions, thus with a detection delay and a POD_0_ gap compared to the onset of HR-like reactions amounting to 18 days and 4.9 mmol O_3_ m^−2^ s^−1^. Similarly, risk assessment studies using visible injury markers rather target the late and final structural evolution of responses to O_3_ stress in foliage, with possible interspecific variation, instead of the injury appearance in foliage.

### Leaf Position Dependency of Responses to O_3_ Stress

Reductions in leaf gas exchanges or the development of HR-like lesions and visible symptoms at the tenth vs. third leaf position occurred later and for larger POD_0_. Given the higher stomatal conductance and POD_0_ in younger leaves, their greater physiological activity and lower levels of injury suggest higher O_3_ tolerance, while a contribution by enhanced stomatal closure can be excluded. This finding is confirmed by similar reports on enhanced O_3_ tolerance in maturing leaves (Reich, 1983; Paakkonen et al., 1996; Strohm et al., 2002; Bagard et al., 2008; Zhang et al., 2010; Guerrero et al., 2013). This may be related to sink functional properties and larger resource availability for defense and repair (Coleman, 1986). Resource availability in young leaves could be increased by the supply of nutrients (such as nitrogen, potassium, and phosphorus) coming from senescent leaves (Maillard et al., 2015; Have et al., 2017). Hence, the concentration of phenolics with antioxidant properties and other antioxidative capacities decline during the sink-to-source transition in maturing foliage, thus increasing leaf susceptibility to oxidative stress (Coleman, 1986; Strohm et al., 2002; Blokhina and Fagerstedt, 2006; Bellini and De Tullio, 2019). However, and in contrast to older foliage, the development of HR-like lesions as a function of POD_0_ at the tenth leaf position depended on the O_3_ treatment, with a higher O_3_ tolerance in the 80 ppb O_3_ treatment. Given the high O_3_ dose in maturing leaves, this finding highlights the importance of the O_3_ absorption rate given the saturation of the antioxidative system. The higher O_3_ tolerance in younger vs. older foliage was further confirmed by their still comparable leaf percentage areas showing HR-like lesions in the 100 ppb O_3_ treatment despite 1.8 times higher POD_0_ at the tenth leaf position.

### Reaction Gradient in Foliage Concerning Critical O_3_ Levels

In our experiment, the current CL (POD_Y_SPEC_ for beech and birch = 5.2 mmol O_3_ m^−2^; Mills et al., 2017) was equivalent to a POD_0_ of 5.7 mmol O_3_ m^−2^. By the end of exposure, this CL had thus been exceeded by 1.54 and 2.35 times at the third and tenth leaf positions, respectively. If any impairment of tree morphology and biomass was still lacking, reductions in leaf gas exchange, development of structural injury, and the emergence of visible symptoms at the third leaf position had already been observed for O_3_ dose, amounting to 0.82, 0.69, and 1.46 times the current CL, respectively. At the tenth leaf position, these responses were detected for POD_Y_SPEC_ 1.83, 1.18, and 2.32 times above CL. These findings highlight the high dependency of sensitivity evaluations on the selected parameters and scale of observation. They also outline the within-tree gradient of sensitivity to O_3_ stress, given the large size of such organisms, which, as a result, complicates O_3_ risk assessment. They finally indicate that below CL, significant effects in the foliage of trees, such as in the impairment of leaf physiology and development of microscopic necrosis in extended parts of mesophyll, can be expected. These responses may already contribute to reduced carbon uptake and storage in foliage and other tree organs before reaching CL thresholds.

## Conclusion

In this study, we characterized the dynamics of physiological, structural, and morphological responses to two levels of O_3_ exposure and as a function of time, POD_0_ and leaf position, in fully controlled conditions. We observed contrasting dynamics, monotonic or sigmoidal-like, as a function of plant responses but irrespective of leaf position, before any visible symptoms and effects on the gross morphology of trees. The first microscopic necrosis developed weeks before the appearance of visible symptoms and at half the O_3_ dose. Concerning experimental hypotheses (H), the sequential development and distinct dynamics of physiological, structural, and morphological responses to O_3_ stress was confirmed (confirmation of H1); both HR-like and ACS responses were elicited, the former occurring first (confirmation of H2a, rejection of H2b). When expressed as a function of POD_0_, leaf responses did not depend on the O_3_ treatment (confirmation of H3a), except for the development of structural injury that depended on the O_3_ absorption rates in younger foliage (partial rejection of H3b). Finally, response dynamics were strongly related to leaf age as a function of time or POD_0_, showing delay in younger foliage (confirmation of H4). This study thus sheds light on the syndrome of early reactions to O_3_ stress and disentangles the specific dynamics of distinct but co-occurring plant responses, before CL exceedance. The resulting variety of symptoms, as observed by the end of the experiment, provides an exemplary experimental demonstration for integrative injury display, as found in the field late in summer. Given ACS and HR-like timing inversion, compared to field conditions, the ontogenetical and environmental drivers also appear to have a prevailing effect over the sensitivity of affected markers, regarding the timing and dynamics of each cellular response. Whatever the exact sequence order of early reactions to O_3_ stress below CL – in the field or controlled conditions, they will modify key foliage properties. Such impacts can be relevant for some, e.g., biodiversity ecosystem services before those of economic significance – i.e., wood production, as targeted by flux-based CL.

## Data Availability Statement

The raw data supporting the conclusions of this article will be made available by the authors, without undue reservation.

## Author Contributions

BT, YJ, MC, and PV: conception or design of the work and final approval of the version to be published. BT: data collection. BT, PV, AG, DT, YJ, and MC: data analysis and interpretation. BT and PV: drafting the article. BT, PV, AG, DT, YJ, MC, and MS: critical revision of the article. All authors contributed to the article and approved the submitted version.

### Conflict of Interest

The authors declare that the research was conducted in the absence of any commercial or financial relationships that could be construed as a potential conflict of interest.
